# Toward structured abdominal examination training using augmented reality

**DOI:** 10.1007/s11548-024-03311-y

**Published:** 2025-01-04

**Authors:** Lovis Schwenderling, Laura Isabel Hanke, Undine Holst, Florentine Huettl, Fabian Joeres, Tobias Huber, Christian Hansen

**Affiliations:** 1 Faculty of Computer Science and Research Campus STIMULATE, Otto-von-Guericke University of Magdeburg, Magdeburg, Germany; 2 Clinic for General, Visceral and Transplant Surgery, University Medical Center of the Johannes Gutenberg University Mainz, Mainz, Germany

**Keywords:** Mixed reality, Augmented reality, Human–computer interaction, Visualization, Surgical training, Medical education

## Abstract

**Purpose:**

Structured abdominal examination is an essential part of the medical curriculum and surgical training, requiring a blend of theory and practice from trainees. Current training methods, however, often do not provide adequate engagement, fail to address individual learning needs or do not cover rare diseases.

**Methods:**

In this work, an application for structured **A**bdominal **E**xamination **T**raining using **A**ugmented **R**eality (AETAR) is presented. Required theoretical knowledge is displayed step by step via virtual indicators directly on the associated body regions. Exercises facilitate building up the routine in performing the examination. AETAR was evaluated in an exploratory user study with medical students (*n*=12) and teaching surgeons (*n*=2).

**Results:**

Learning with AETAR was described as fun and beneficial. Usability (SUS=73) and rated suitability for teaching were promising. All students improved in a knowledge test and felt more confident with the abdominal examination. Shortcomings were identified in the area of interaction, especially in teaching examination-specific movements.

**Conclusion:**

AETAR represents a first approach to structured abdominal examination training using augmented reality. The application demonstrates the potential to improve educational outcomes for medical students and provides an important foundation for future research and development in digital medical education.

**Supplementary Information:**

The online version contains supplementary material available at 10.1007/s11548-024-03311-y.

## Introduction

Structured abdominal examination is an important routine procedure used in many clinical settings and an important part of surgical training. Medical students need to learn a variety of symptoms and indicators in addition to acquiring the skills and practice. However, there is often a lack of opportunities for regular practice in clinical settings and support for individual practice [1].

Augmented reality (AR) in education and training has demonstrated significant benefits, including improved comprehension and increased motivation for learning [2]. AR can provide cues to the user directly at the point of action without obstructing the perception of the environment, thus supporting cognitive processing [3]. Many studies comparing AR to traditional teaching methods report equal or lower cognitive load, as well as higher performance of the students [4]. AR has particular advantages for training in risk situations that are difficult to simulate in reality, e.g., in healthcare [5].

In this work, we present AETAR (Abdominal Examination Training in Augmented Reality), an AR-based educational tool for teaching abdominal examination techniques. AETAR integrates multiple examination procedures into a unified training platform.

## Related work

AR has been shown to be beneficial in medical education, enhancing the learning process [6] and showing consistent and promising results in knowledge tests [7]. Significant improvements are often seen in student satisfaction and engagement [8]. Students find the 3D visualization properties [9] and content interactivity [10] particularly important and report higher motivation and enjoyment when learning in an AR environment [11]. A recent meta-analysis [12] indicates improvements in response, performance, knowledge and skill through the usage of adequate AR.

While there is no application to date that teaches the entire abdominal examination, there are related works that address the individual sub-steps. In a pilot AR project, Sen et al. [13] visualized cutaneous signs of diseases of the gastrointestinal systems on a phantom with image markers. Using a mobile phone, markers could be scanned and symptoms could be viewed on the screen. Additionally, a magic lens to visualize organ positions during palpation was provided. However, no evaluation of the system was performed. With focus on pulmonary instead of abdominal examination, Pieterse et al. [14] presented an AR application to support lung auscultation. Various example cases, which included auditory examples and associated 3D models, were developed. In an evaluation, students stated that they had gained a better understanding of the respective diseases through the application. Sherstyuk et al. [15] augmented the percussion step of the abdominal examination using a phantom with tactile sensors and hand tracking. Auditory feedback was given based on the location that was tapped, using knocking and sounds indicating that the patient is experiencing pain. No visual augmentation was provided. Asadipour et al. [16] presented a palpation training environment using pressure sensors at both hands. Screen-based feedback was given on finger position and pressure intensity. Participants using the system performed significantly better compared to a control group taught by traditional methods. Muangpoon et al. [17] present a training application for the rectal portion of the abdominal examination. Using the HoloLens 1st generation and a medical training phantom, they visualized the finger of the examiner as well as anatomical structures surrounding the anal canal to support understanding of finger maneuvers. In their study, the system was found to be useful for teaching and learning by medical students and clinicians. Limbs&Things Inc. (USA) presented a mobile AR application^1^, accompanying their medical training phantom for abdomen examinations. Overlay views of inner structures and example videos are included to support understanding. However, no structured guidance throughout the procedure is provided.

Although AR has generally been shown to be beneficial both in teaching sub-steps of the examination and in teaching tasks with both practical and theoretical components, a unified training platform for the entire abdominal examination, not just individual sub-steps, has not yet been presented.

## Material and methods

The hardware and software configuration, the training application itself and the evaluation of AETAR are described in this section.

### Setup

AETAR is based on an anatomically correct male torso training phantom that is already used in medical training (Abdominal Examination Trainer by Limbs&Things). This is used for teaching the abdominal examination. It provides a flexible plug-in system for different organs allowing for the simulation of various diseases. A small fat pad and no organ variations were used to simulate a healthy human being. For AR representation, a high-resolution 3D model of the medical training phantom was created using tools based on structured-light imaging provided by the ProAV suite of ProjectionTools (domeprojections.com GmbH, Germany). The HoloLens 2nd generation (HL2, Microsoft Corporation, USA) was selected to create the AR environment as it provides freedom of movement and is widely used for AR in the medical context [18]. It was also used for its eye, hand and marker tracking abilities as well as optical see through design to facilitate usage in a clinical environment. Unity (Unity Technologies, USA) was used for the implementation. Interaction and interactive elements were implemented using the Mixed Reality Toolkit V2^2^ in Unity. Image tracking for registration of the medical training phantom was implemented with Vuforia AR SDK (PTC Inc, USA) integrated in Unity.

**Fig. 1:**
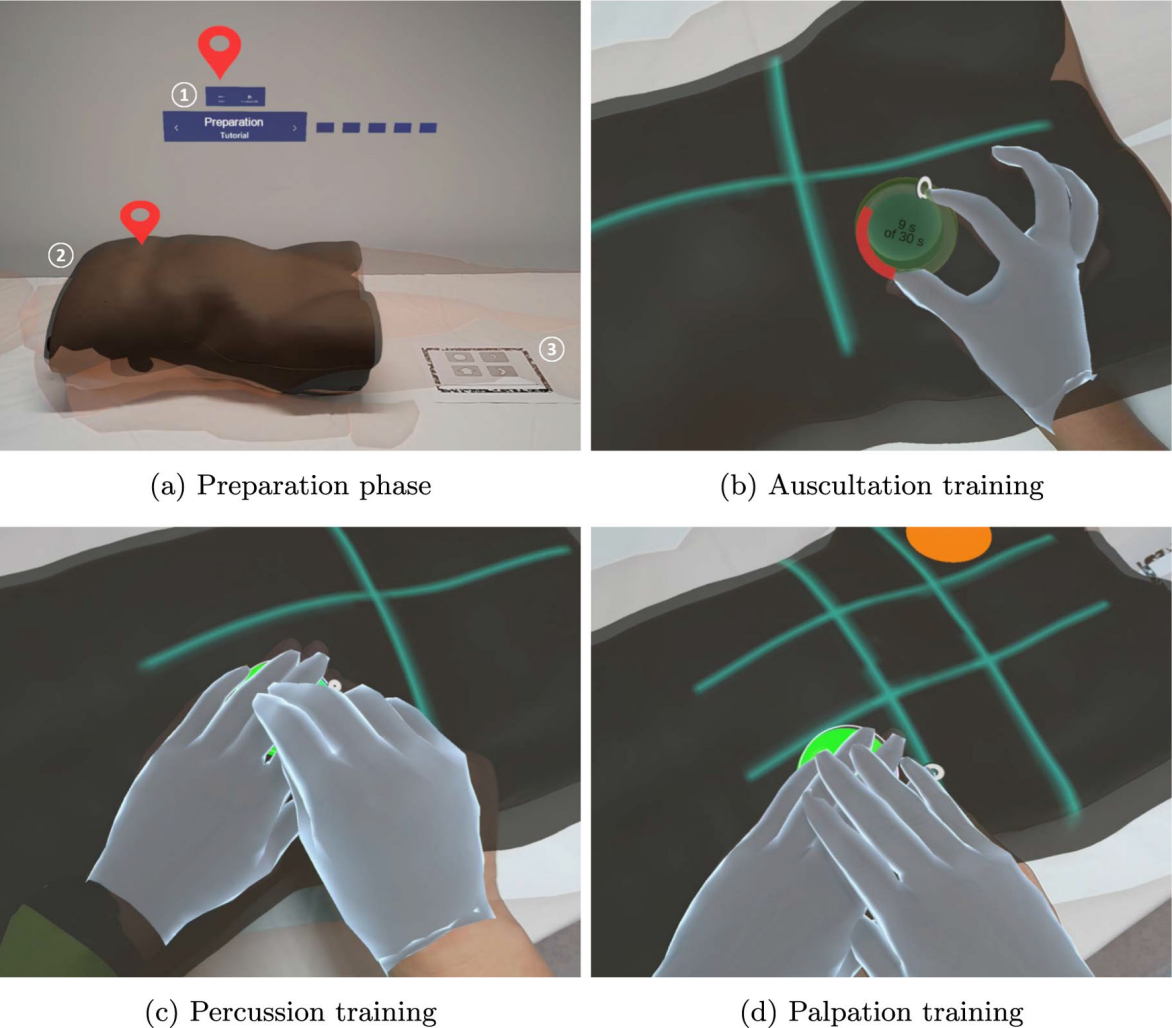
Layout of AETAR in the preparation phase **a**: 1 - *floating menu*, 2 - medical training phantom and the *AR phantom*, 3 - *registration marker* (3). Additionally, two red indicators are displayed to access information. For auscultation **b**, the AR stethoscope had to be placed on the marker, upon which a circulating timer of 30 s was displayed. Guiding lines , subdividing the abdomen into quadrants, are displayed to visualize the examinations structure. These were also used for the percussion **c**. Here, the circular marker was intended to be pressed like a button with one hand, while performing a tapping movement with the other. For the palpation **d**, the abdomen was divided into nine regions, also represented by guiding lines. An orange circle visualized the center of pain. The circular marker in this case was intended to be pressed with both hands at once. The smooth virtual outline of the hands was added for visibility in the photos. Further impressions are available in the supplementary video.

### Concept development

AETAR was developed in cooperation with the University Medical Center of the Johannes Gutenberg University Mainz. First, a detailed flowchart of the abdominal examination was created based on medical textbooks [19–21] and instructional videos provided by the clinical partners. To ensure the completeness and correctness of the extracted information, the clinical workflow was then verified with clinicians teaching the examination. The content is consistent with the instructions provided at the University Medical Center of the Johannes Gutenberg University Mainz, so small differences may occur with other institutions.

Based on the documented workflow, areas where AR can support learners were identified. Together with teaching clinicians, AR concepts were defined and validated with regard to their conceptual suitability for medical teaching. A program flowchart was created from the concepts and the underlying clinical structure and this was verified again with the clinicians. Then, a first prototype was implemented. In a qualitative assessment, this was shown to three physicians and one medical student to obtain initial comments on usability and content appropriateness. Any errors and usability problems that were identified were then corrected, resulting in the application now presented.

### Training application

AETAR is composed around a torso-only training phantom. To create context for symptoms on head and limbs and to generate a more realistic setting, the *AR phantom*, a simplified full body 3D model, is provided. A *floating menu* included a status display and interaction elements to navigate the application. It included forward and back buttons, access to the main menu and tracking controls. A *registration marker* was placed next to the medical training phantom. The layout is displayed in Fig. 1a. Buttons and indicators can be pressed directly or via airtap. A pinch gesture is used to grasp objects.

**Fig. 2:**
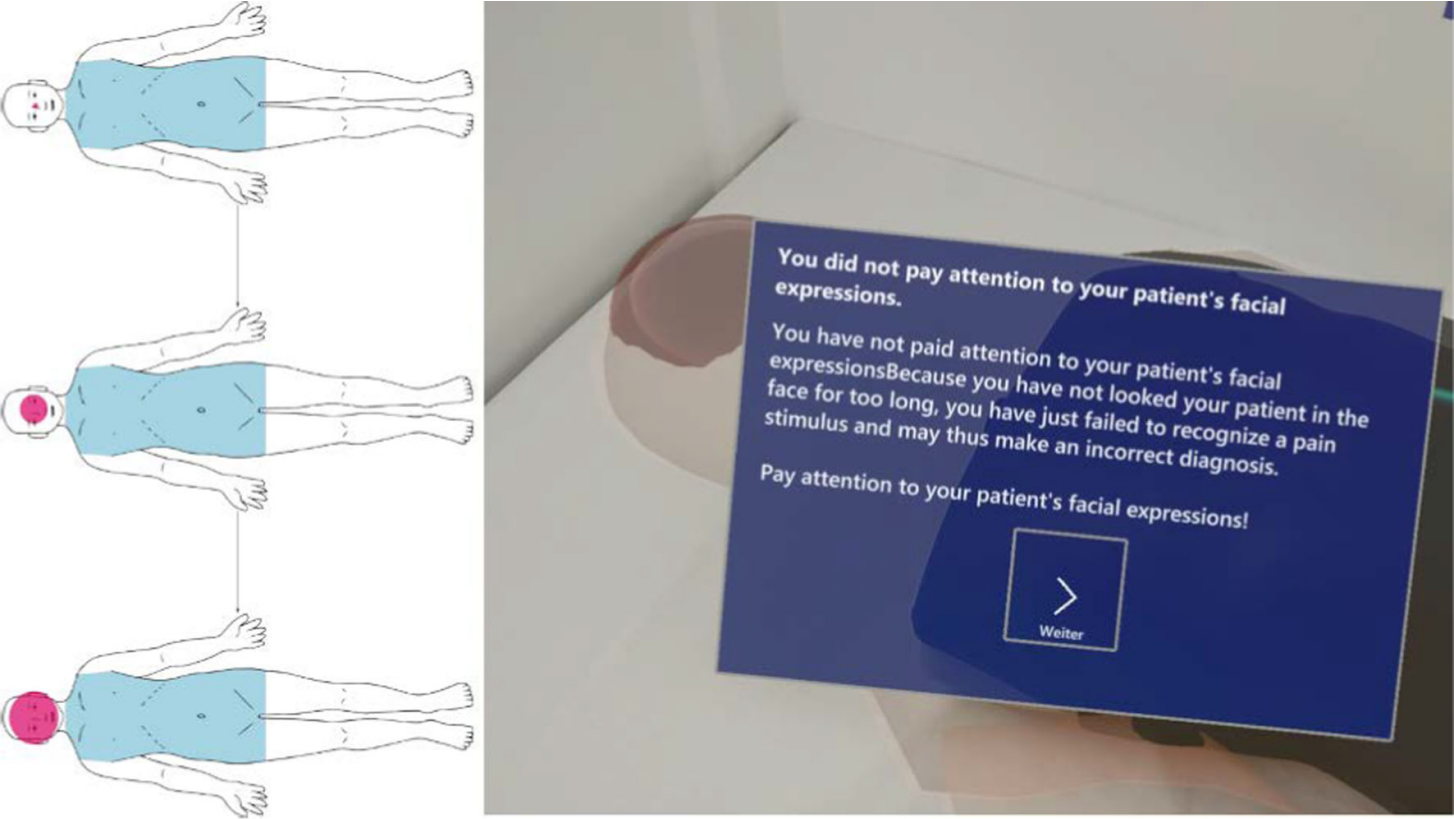
Concept and AR display of the gaze target. The concept on the left shows the AR phantom (black outline), the medical training phantom (blue) and the gaze target (red). The gaze target is intended to teach the user to focus a patients face regularly during percussion and palpation so as not to miss signs of pain. A red balloon was placed on the face, which grew larger over a time period of ten seconds. If the user looked at the face, the gaze target was reset. If the face was ignored for more than ten seconds, an info popup appeared

Teaching content was linked to body regions using *pin indicators* (see Fig. 1a). Indicators were presented in three ways: red, for important information, yellow, for more advanced knowledge not required by the curriculum, and semi-transparent for already selected pins. Triggering an indicator opened a popup (see Fig. 2), which included a short explanation text and, optionally, an image, audio sample or further information accessible by a button. Popups were placed in front of the user at a distance of about 60 cm and followed larger head movements. After selecting all indicators, an automatic query was made whether the next step should be started. Users could also navigate to the next step via the floating menu at all times.

***Patient Preparation and Inspection*** The first step of the application was the *Preparation*. It taught how a patient should be positioned for a relaxed abdominal wall. The AR phantom was presented correctly positioned, with important aspects highlighted using indicators. This step was also used to familiarize the user with AETAR by introducing basic interaction elements and the handling of the application.

In the subsequent *Inspection* step, external signs of abdominal diseases were presented on the phantom. Due to the large variety of symptoms to consider, the exploration mode was subdivided into three clusters: deformations, cutaneous signs of diseases, and scars and hematomas.

***Auscultation Training*** In the *Auscultation* step, an *AR stethoscope* was used to teach how to examine the patient’s bowel sounds. For this purpose, the abdomen was divided into quadrants for the examination, for which guiding lines were displayed (see Fig. 1b). To indicate directions for practical examinations, a *circular marker* was used (see Fig. 1b for more details). It was successively displayed in each quadrant to show where the AR stethoscope had to be placed. If a collision detection using Unity capsule colliders indicated correct positioning, a thirty-second timer with a radial loading bar was displayed and normal bowel sounds were played. After learning the process, possible pathological findings were taught and, if available, supported by audio examples.

***Percussion Training*** In the *Percussion* step, the evaluation of tapping sounds for examining the abdomen was taught. The circular marker had to be pressed like a button, guiding through the quadrants marked by guiding lines (see Fig. 1c). In addition, liver size determination, ascites diagnosis and percussion of the flank lines were addressed. A new feature was introduced: the *gaze target* was used to teach users to maintain regular eye contact to check for pain caused by percussion. It required the user to look at the face of the AR phantom at least every ten seconds. To provide feedback and to motivate users to look at the head, a red balloon was displayed that grew larger while the user looked elsewhere (see Fig. 2). When the balloon was focused, it disappeared. If the user did not look at the face in time, a reminder was displayed (see Fig. 2).

***Palpation Training*** In the *Palpation* step, palpation of organ changes and identification of pain points were taught. This was done in two steps: first, general palpation based on the nine abdominal regions (see Fig. 1d) was demonstrated. This was followed by more information on examination strategies for appendicitis and gallbladder evaluation. For the normal palpation, a distinction was made between superficial and deep palpation, in which different degrees of pressure are applied. Starting from the region farthest from the center of pain, users were guided through the examination twice in nine steps, using the circular marker. They were instructed to perform palpation movements on the circle. The movements were not taught or explained in detail, as our clinical partners stated in the workflow analysis that they were already known from other previous courses. The gaze target was also used here. The signs of appendicitis and the gallbladder examination were then presented in the next sub-step, using indicators. Pain points, such as McBurney, were displayed directly on the phantom. Due to the design of the medical training phantom, these were taught theoretically only and there was no practical exercise.

### User study

A mixed-methods exploratory user study was conducted to evaluate the suitability of the application for teaching the abdominal examination.

***Sample Design*** Participants who had already covered the abdominal examination in their studies were invited. As AETAR is intended to be an addition to current teaching, students would be expected to be familiar with the matter. Participants were recruited at the University Medical Center of the Johannes Gutenberg University Mainz.

Twelve students (7f, 5 m) aged 23 to 31 (median = 27) participated in the study. They were in their fourth to sixth year of study (median = 4). Most had previous experience in performing the abdominal examination on patients beyond the lecture, but were rather inexperienced with the medical training phantom. Prior experience with AR was generally nonexistent.

***Variables*** The usability of the application was quantitatively assessed using the system usability scale (SUS) [22]. It was applied after each examination step to evaluate the usability and potential for improvement at each stage. Thus, five subscores were collected: patient preparation ($SUS_{prep}$), inspection ($SUS_{ins}$), auscultation ($SUS_{aus}$), percussion ($SUS_{per}$), and palpation ($SUS_{pal}$). The average value reflected the overall application: $SUS_{AETAR}$.

A *knowledge test* was developed in advance of the study with teaching clinicians to assess the students’ familiarity with the abdominal examination. Six questions about the examination procedure, cutaneous signs of liver diseases, appendicitis signs, auscultation process, and patient preparation were included (see supplementary material). On a *self-assessment* questionnaire, participants were asked to rate their knowledge of the examination in total and of each step, as well as their confidence in performing the examination on the phantom and on a patient on 5-point Likert items ranging from 1 - ”very uncertain/ unconfident” to 5 - ”very certain/ confident”. Both questionnaires were completed twice: once before (pre) and once after (post) going through the application.

Additional qualitative feedback was gathered in a final semi-structured interview. Participants were asked for *general comments* and for feedback in three categories: *interaction*, *application adequacy*, and *teaching perspectives*.

***Procedure*** First, the participants were informed about data protection and the purpose of the study. Written consent and demographic data were then collected. Next, the pre-iterations of the knowledge test and the self-assessment were completed. The participants were asked to put on and adjust the HL2. Eye calibration was performed and the HoloLens Tips app provided by Microsoft was launched to consistently teach interaction with AR content. Then, participants were asked to launch AETAR from the start menu. The basic setup of and interaction with the application was explained. Participants were asked to go through the patient preparation step at their own pace and to voice any comments or questions. After completing the patient preparation, the participants completed the SUS for this step. If there were no further questions about the study process, this procedure was repeated for each subsequent examination step. Any comments made during the study were recorded. Depending on the statement, technical assistance was provided, recommendations were given or further questions were asked. After completing the application, participants were asked to complete the post-iterations of the knowledge test and self-assessment questionnaire. Then, the semi-structured interview was conducted. On average, one session lasted one and a half hours.

**Fig. 3:**
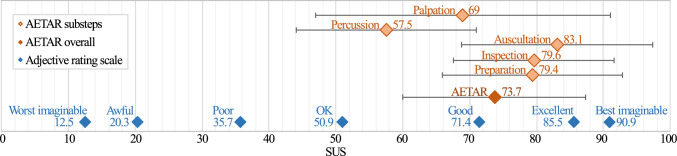
Visualization of the SUS results (diamond = mean, error bars = standard deviation) with the adjective SUS rating scale based on Bangor et al. [23]

### Expert interviews

Complementary to the students’ perspectives, the correctness and appropriateness of the application for medical teaching were evaluated with expert interviews. Two physicians who were not directly involved in the development process participated. One of them already had previous experience with AR, the other none at all. Both perform the abdominal examination regularly and know the challenges of teaching it.

After a brief introduction to interaction with the HL2, they were then asked to complete AETAR at their own pace, making comments and suggestions as they went. Technical assistance was provided if necessary. Additionally, questions were asked about the correctness of the information presented, its use in education, and the presentation of the teaching content. Finally, a short semi-structured interview was conducted, with a focus on the suitability for teaching and the teaching setting.

## Results

**Fig. 4:**
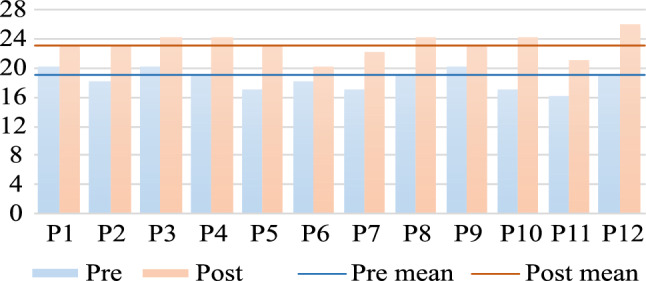
Visualization of the knowledge test scores for each participant before and after AETAR was completed

**Fig. 5:**
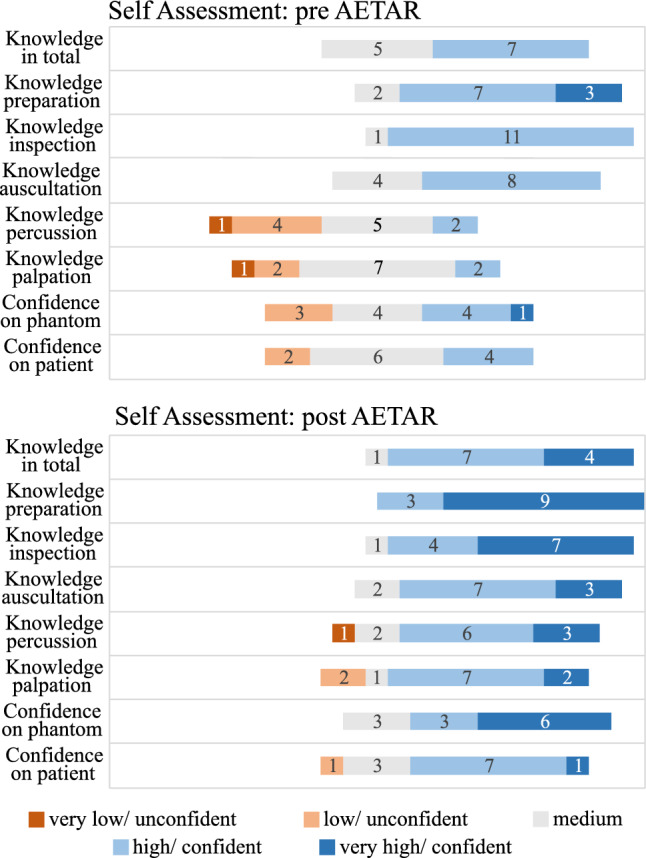
Visualization of the self-assessment of the participants knowledge and confidence with the abdominal examination before (upper plot) and after (lower plot) using AETAR. The distribution of the six participants for each of the Likert items is shown

SUS scores were calculated according to the definition of Brooke [22] and are displayed in Fig. 3. The knowledge test scores were determined using a sample solution defined by teaching clinicians. The scores of each participant, as well as the pre- and post-average, are displayed in Fig. 4. For self-assessment, the distribution on the 5-point Likert scale pre- and post-AETAR is illustrated in Fig. 5. The qualitative feedback of the students is summarized in Table 2. Only statements made by more than one person were included, with similar comments clustered. The results for the teachers are listed in Table 1.

**Table 1:** Summary of the interview results of the teachers. Advantages and disadvantages in terms of teaching setting and suitability of AETAR that were mentioned are listed

**Advantages**	**Disadvantages**
$\bullet$ Good tool to review and repeat the process individually	$\bullet$ Correctness of the execution is not checked
$\bullet$ Encourages students to work independently	$\bullet$ More feedback on the current progress should be provided to the students
$\bullet$ Spatially correct placement and practical use of guiding lines and info	$\bullet$ No correct movements are required for the percussion
$\bullet$ Gaze target reminds students that they have to pay attention to the patient	$\bullet$ Student progress is not transparent to instructors; questions may be difficult to answer as a result
$\bullet$ Easier because no patients need to be trained/informed

**Table 2:** Summary of the interview results of the students. Statements from more than one participant were clustered and listed

Positive	Negative
*General comments*	
$\bullet$ Listening examples are helpful	$\bullet$ Popups are badly positioned
$\bullet$ Fun and motivating, interesting	$\bullet$ Field of view (HL2) is too small
	$\bullet$ Needed head posture is unnatural
*Interaction*	
	$\bullet$ Hand interaction is intuitive and preferred over other possible forms of interaction
$\bullet$ Buttons are hard to press at start	$\bullet$ The gaze target is often not recognized correctly and hinders hand interaction
*Application adequacy*	
$\bullet$ Consistent with teaching content	$\bullet$ Proper percussion and palpation movements are not taught or required to perform
	$\bullet$ Spatial relationships are especially helpful with the skin inspection
*Teaching perspectives*	
$\bullet$ Would be a good addition to the current teaching	$\bullet$ Too many technical errors occur to allow for individual usage without technical support
$\bullet$ Can be used in student-organized practice labs	$\bullet$ Can’t replace practice on patients

All participants improved in the knowledge test after going through AETAR (see Fig. 4. They showed an average improvement of 4 points (pre: $19.1\pm 2.9$ points, post: $23.1\pm 1.5$ points). This was mainly caused by naming more cutaneous signs of liver diseases. Participant feedback particularly emphasizes the increased understanding of the distribution of symptoms throughout the patient’s body.

Overall, participants rated their knowledge and confidence in performing after AETAR as higher than before (see Fig. 5). Although the training only took place on the medical phantom, the confidence with the patient also improved. This may be caused by knowledge improvement overall.

The usability was rated as good (see Fig. 3). Aspects that affected the usability were issues with hand interaction and occasional display problems. Additionally, the positioning of pop-ups was often criticized, causing difficulties with reading and interaction. The best SUS value was obtained during auscultation, where participants especially liked the auditory examples. The percussion was rated as the least usable step. Participants described the circular marker as irritating and unintuitive. Additionally, many users experienced technical problems with the gaze target, i.e., that looking at the face was not recognized correctly. However, participant self-assessment and feedback of the teaching clinicians also indicated that most students struggle with these stages in general as these are hard to learn from textbooks or on training phantoms.

## Discussion

Regarding the suitability of the application for education, students and teachers agreed about the potential and benefits, but have also noted that there is still room for improvement in the area of interaction and technical errors. This suggests that usability in general should be improved first before AETAR is suitable for wider use or investigating the teaching effectiveness specifically. Feedback from other institutions could also enhance the general suitability for training. Furthermore, the addition of further teaching content is of interest. With the AR tool of the Limbs&Things training phantom, a deeper understanding is aspired by displaying the organs and the effects of examination methods. This could also support AETAR, but care should be taken that it does not interfere with the examination process. Students using AETAR also emphasized the importance of audio samples. This is in line with related work [14, 15] and should therefore be extended in the future.

Ergonomic problems encountered, such as a too small a field of view requiring lots of unnatural head movements, are known issues in other AR scenarios [24]. Here, the use of other XR displays could be considered in the long term. This may also help with hand tracking issues. Furthermore, a complementary interaction method, e.g., voice interaction, and further visual and/or auditory feedback for interactions can be offered in the future. However, this was not included in the current prototype as this could lead to disruptions in the intended teaching use. The lack of comprehensibility of learning progress for teachers was criticized during evaluation. Related work has presented models that analyze learning progress and offer support options [25]. While this application was developed with teaching physicians, further iterations should aim to evaluate and continue with a wider group of teachers.

A self-designed knowledge test was designed to evaluate the knowledge gain in this study. In medical teaching, the evaluation takes place within the framework of a practical examination as well as on case studies. In future studies this should be considered and adopted for the evaluation of AETAR. However, no participant reached the full score in the knowledge test in either iteration. Therefore, the difficulty level of the test was believed to be well chosen and to be representative as an indicator of the teaching potential of AETAR. Improvements in the knowledge test may be caused by the repetition of the material itself by using the application. Future studies should consider this with a control group spending the same amount of time using textbooks or alternative teaching formats, as is more common in AR anatomy applications, for example [26]. Furthermore, an evaluation of the long-term knowledge gain should also be taken into account to provide insights beyond short-term memory. Self-assessment questionnaires and post-study interviews are susceptible to social desirability bias. However, the subjective qualitative and quantitative results are supported by objective knowledge test improvement. Nevertheless, in future studies, more emphasis should be placed on objective measurements, such as knowledge tests or examination performance.

Participants volunteered to participate and received no compensation for doing so. Thus, only students with high motivation and interest may have registered. This may affect the representativeness of the results, especially in terms of learning outcomes. Another study also found differences in the appreciation of AR in relation to gender and specialization in studies [27]. While the impact on the suitability and usability evaluation of this work was likely small, future studies should randomize participants to avoid bias.

Two further areas for improvement were identified: the gaze target and the realism of the examination. For the gaze target, aspects from attention guidance [28] can be integrated in order to achieve a lasting learning effect and familiarization. Overall, a more realistic design of the AR phantom is important, both for the gaze target and for the entire immersion into the examination procedure. Sen et al. [13] showed the skin signs directly on the skin. This was also requested in the feedback for AETAR and represents important future work. Furthermore, inclusion of practical training of all examination methods is an important point of improvement. Tactile sensors, as used in related work [15, 16], might be superior to the hand tracking provided by the HL2, but would require additional and possibly cumbersome hardware.

## Conclusion

This work presented AETAR, an application to support the individual learning of the abdominal examination in medical school. Using the HL2, users are guided through the examination process, possible symptoms and context information.

An exploratory user study with medical students and interviews with teaching clinicians were conducted. SUS scores and pre- and post-iterations of a knowledge test as well as a self-assessment of knowledge and confidence were acquired. Results showed an improvement in the knowledge test for all participants, as well as higher self-perceived knowledge and confidence. The usability was rated as good whereby the percussion showed the most room for improvement.

While the implementation of some examination steps was criticized as too unrealistic and thus not beneficial, both, teachers and students, found the application overall to be suitable for teaching. AETAR and comparable future applications can thus represent a key element in supporting the self-learning skills of medical students.

## Supplementary Information

Below is the link to the electronic supplementary material.

Supplementary file 1 (mp4 246290 KB)

Supplementary file 2 (pdf 545 KB)

## Footnotes

1 https://limbsandthings.com/global/products/60000-abdominal-examination-trainer-light-skin-tone Retrieved 28 October 2024.

2 https://github.com/Microsoft/MixedRealityToolkit-Unity Retrieved 22 March 2024.
