# Supplementary material for: Toward structured abdominal examination training using augmented reality
Source: Int J Comput Assist Radiol Surg. 2025 Jan 4;20(5):949–58. doi: 10.1007/s11548-024-03311-y (PMC12055959; doi:10.1007/s11548-024-03311-y)
Supplement: Supplementary file 2 — (pdf 545 KB) [file 11548_2024_3311_MOESM2_ESM.pdf]

Supplementary Material: Knowledge Test

Article title: Towards Structured Abdominal Examination Training using Augmented Reality

Journal name: International Journal of Computer Assisted Radiology and Surgery

Authors: Lovis Schwenderling[1], Laura Isabel Hanke[2], Undine Holst[1], Florentine Huettl[2], Fabian Joeres[1], Tobias Huber[2], Christian Hansen[1]

1 - Faculty of Computer Science and Research Campus STIMULATE, Otto-von-Guericke University of Magdeburg, Germany.

2 - Clinic for General, Visceral and Transplant Surgery, University Medical Center of the Johannes Gutenberg University Mainz, Germany.

Corresponding author: Christian Hansen, hansen@isg.cs.uni-magdeburg.de

Knowledge Test Abdominal Examination

1. Name the individual steps of the structured abdominal examination in the correct order. (1 point / correct step, 1 point for correct sequence)

- Inspection
- Auscultation
- Percussion
- Palpation

2. Name all the liver signs you know. (1 point per liver skin sign)

- Spider naevi
- Gynecomastia
- Icterus
- Palmar erythema
- Caput medusae
- Abdominal baldness
- Petechiae
- White nails
- Striae
- Lacquer lip/tongue

3. Name all the signs typical of appendicitis that you know. (1 point/sign)

- Psoas
- McBurney
- Lanz
- Blumberg
- Rovsing

4. Which appendicitis sign is considered obsolete and is no longer tested? (1 point)

- Rovsing

5. How long should you auscultate over each quadrant? (1 point)

30 seconds to 1 minute, in the absence of bowel sounds up to 3 minutes /quadrant

6. How should the patient be positioned (arms, legs, head position) and why? (one point for the positional instructions and one point for the explanation)

- Arms next to the body, not crossed
- Legs stretched out next to each other, not crossed, if necessary with leg roll
- Head relaxed and laid down, if necessary with a pillow
- Explanation: the abdominal wall need to be relaxed and the abdomen and groin have to be well accessible
